# L-Lysine-Modified pNIPAm-co-GMA Copolymer Hydrogel for pH- and Temperature-Responsive Drug Delivery and Fluorescence Imaging Applications

**DOI:** 10.3390/gels9050363

**Published:** 2023-04-25

**Authors:** Madhappan Santhamoorthy, Ramkumar Vanaraj, Kokila Thirupathi, Selvakumari Ulagesan, Taek-Jeong Nam, Thi Tuong Vy Phan, Seong-Cheol Kim

**Affiliations:** 1 School of Chemical Engineering, Yeungnam University, Gyeongsan 38541, Republic of Korea; 2 Department of Physics, Government Arts and Science College for Women, Karimangalam 635111, Dharmapuri, Tamil Nadu, India; 3 Division of Fisheries Life Sciences, Pukyong National University, Nam-gu, Busan 48513, Republic of Korea; 4 Institute of Fisheries Sciences, Pukyong National University, Gijang-gun, Busan 46041, Republic of Korea; 5 Center for Advanced Chemistry, Institute of Research and Development, Duy Tan University, 03 Quang Trung, Hai Chau, Danang 550000, Vietnam; 6 Faculty of Environmental and Chemical Engineering, Duy Tan University, 03 Quang Trung, Hai Chau, Danang 550000, Vietnam

**Keywords:** pH and temperature stimuli, hydrogel, drug delivery, fluorescence imaging, biocompatibility

## Abstract

The development of dual-stimuli-responsive hydrogels attracts much research interest owing to its unique stimuli-responsive characteristics. In this study, a poly-N-isopropyl acrylamide-co-glycidyl methacrylate-based copolymer was synthesized by incorporating N-isopropyl acrylamide (NIPAm) and a glycidyl methacrylate (GMA) monomer. The synthesized copolymer, pNIPAm-co-GMA was further modified with L-lysine (Lys) functional units and further conjugated with fluorescent isothiocyanate (FITC) to produce a fluorescent copolymer pNIPAAm-co-GMA-Lys hydrogel (HG). The in vitro drug loading and dual pH- and temperature-stimuli-responsive drug release behavior of the pNIPAAm-co-GMA-Lys HG was investigated at different pH (pH 7.4, 6.2, and 4.0) and temperature (25 °C, 37 °C, and 45 °C) conditions, respectively, using curcumin (Cur) as a model anticancer drug. The Cur drug-loaded pNIPAAm-co-GMA-Lys/Cur HG showed a relatively slow drug release behavior at a physiological pH (pH 7.4) and low temperature (25 °C) condition, whereas enhanced drug release was achieved at acidic pH (pH 6.2 and 4.0) and higher temperature (37 °C and 45 °C) conditions. Furthermore, the in vitro biocompatibility and intracellular fluorescence imaging were examined using the MDA-MB-231 cell line. Therefore, we demonstrate that the synthesized pNIPAAm-co-GMA-Lys HG system with temperature- and pH-stimuli-responsive features could be promising for various applications in biomedical fields, including drug delivery, gene delivery, tissue engineering, diagnosis, antibacterial/antifouling material, and implantable devices.

## 1. Introduction

Poly(N-isopropylacrylamide) (pNIPAAm) is a well-familiar thermoresponsive polymer and its lower critical solution temperature (LCST) is observed at 32 °C [1]. Owing to the presence of hydrophilic amide and hydrophobic propyl groups, the pNIPAAm polymer exhibits a reversible linear-to-globule phase transition at a lower critical solution temperature of 32 °C. At below the LCST (<32 °C), the pNIPAAm undergoes swelling by absorbing more water, whereas at a higher LCST (>32 °C) the polymer becomes hydrophobic due to the interaction of the hydrophobic methyl groups in the polymer chains. As a result, the polymer chains undergo shrinkage and release the loaded cargo. For controlled drug release applications, the NIPAAm is copolymerized with another appropriate comonomer to increase the drug loading and release efficiency [2]. Epoxy groups containing copolymers are recognized as versatile polymeric materials for various applications. Poly(glycidyl methacrylate) is considered to be an interesting polymer with epoxy side groups. Glycidyl methacrylate (GMA) is a considerably hydrophobic and non-toxic monomer, a highly reactive, cheaper monomer, which can be easily copolymerized with reliable comonomers to produce multifunctional polymers. Due to the high reactivity of the pendant epoxy groups with amine or thiol groups, it can be utilized further to modify appropriate functional units and improve the applicability of the resulting copolymers [3,4].

A stimuli-responsive drug delivery system that enables releasing the therapeutic drugs to the target sites in a controlled manner has attracted much attention [5,6]. In this aspect, a range of materials including polymeric nanoparticles, hydrogels, inorganic nanocarriers, liposomes, etc., have been applied for controlled drug delivery applications [7,8,9,10,11]. Among these, stimuli-responsive copolymer-based hydrogels that can respond to internal and/or external stimuli, including pH, temperature, light, and magnetic fields, are extensively studied [12,13,14,15,16]. Particularly, temperature-stimuli-responsive copolymer hydrogels that are sensitive to temperature variations attracted much research interest in various fields, including drug delivery, wound dressing, and tissue engineering [17,18,19,20,21]. A range of internal or external stimuli such as pH, temperature, enzyme, magnetic field, etc., have successfully been utilized as stimuli to achieve controlled drug delivery. Among them, temperature and intracellular pH are considered effective stimuli for therapeutic applications [22,23]. The combined dual stimuli, specifically temperature/pH, temperature/enzyme, pH/magnetic field, etc., have been considered much more important owing to their combined effects [24,25]. The development of dual-stimuli-responsive controlled drug delivery systems is considerably important for target site-specific drug delivery. In this aspect, temperature- and pH-responsive copolymer hydrogels are gaining popularity in drug delivery. The dual-stimuli-responsive copolymers are synthesized by copolymerizing two or more reliable monomers in which one segment is responsive to temperature and the other segment contains amine (–NH_2_) or carboxylic acid (–COOH) functional groups. These amine or carboxylic acid groups act as drug-binding sites for loading therapeutic agents, and pH-stimuli-responsive drug release can be accomplished by the protonation or deprotonation of these functionalities [26,27]. The copolymerization of a NIPAAm monomer with pH-responsive amine or carboxylic acid-containing monomers results in dual pH- and temperature-responsive copolymer hydrogels.

Recently, the development of fluorescent-based copolymer hydrogels is considered significant in medical applications. Because of its fluorescent characteristics, copolymer hydrogels are utilized for bioimaging-based traceable and target drug delivery and fluorescence-based biosensing of specific analytes [28]. Owing to the combined pH and temperature responsiveness and fluorescent imaging characteristics in a single system, the copolymer hydrogels are applied for both drug delivery and fluorescence-based bioimaging applications [29]. The pH difference between the normal tissues (pH 7.4) and tumor tissues (pH < 7.4) makes the system pH responsive. The loaded drugs retain the hydrogels at physiological pH conditions and release them under acidic pH environments. Furthermore, the temperature stimuli enhance the drug diffusion from the hydrogel system.

In this paper, we propose the synthesis of a temperature- and pH-responsive pNIPAAm-based copolymer by integrating it with glycidyl methacrylate (GMA) and further modifying it with L-lysine units to produce a pNIPAAm-co-GMA-Lys copolymer hydrogel (HG) system. In the prepared pNIPAAm-co-GMA-Lys HG, the integrated pNIPAAm segment is responsible for temperature-responsive swelling–deswelling by a sharp phase transition. Similarly, the GMA segment with L-lysine functional units acts as the pH-responsive drug-binding sites. Furthermore, the fluorescein isothiocyanate (FITC) was conjugated with the copolymer pNIPAAm-co-GMA-Lys HG to extend the applicability of the pNIPAAm-co-GMA-Lys HG for bioimaging application. The synthesized copolymer pNIPAAm-co-GMA-Lys HG was characterized by different instrumental techniques, including FTIR, ^1^H NMR, SEM, zeta potential, and particle size analysis. The drug loading and dual temperature- and pH-responsive drug release behavior of the copolymer pNIPAAm-co-GMA-Lys HG was evaluated at different temperatures (25 °C, 37 °C, and 45 °C); different pH (pH 7.4, 6.2, and 4.0); and combined temperature and pH (45 °C/pH 7.4, 45 °C/pH 6.2, and 45 °C/pH 4.0) conditions, respectively, by using Cur as a model drug. Furthermore, the in vitro biocompatibility and fluorescence behavior of the copolymer pNIPAAm-co-GMA-Lys HG was examined on the MDA-MB-231 cell line.

## 2. Results and Discussion

### 2.1. Characterization of pNIPAAm-co-GMA and pNIPAAm-co-GMA-Lys HG Samples

**Figure 1:**
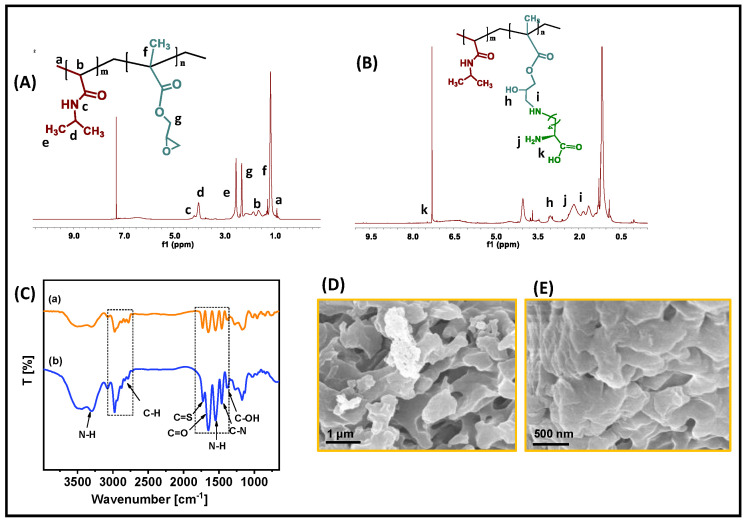
^1^H NMR spectra of (**A**) pNIPAAm-co-GMA copolymer and (**B**) pNIPAAm-co-GMA-Lys HG; (**C**) FTIR analysis of (a) pNIPAAm-co-GMA copolymer and (b) pNIPAAm-co-GMA-Lys HG samples. SEM images of the (**D**) pNIPAAm-co-GMA copolymer; (**E**) pNIPAAm-co-GMA-Lys HG.

The ^1^H NMR spectrum investigation confirmed the structure of the produced pNIPAAm-co-GMA copolymer sample. The ^1^H NMR spectra of the hydrogel system in a CDCl_3_ solvent are shown in Figure 1A. All the relevant proton signals for the existing pNIPAAm and GMA segments in the pNIPAAm-co-GMA copolymer are presented in Figure 1A. The resonance peaks for the methyl (–CH_3_) and –OCH_2_- groups appeared at δ1.2 ppm and δ2.4 ppm, respectively. The resonance signal at δ4.5 and δ4.7 ppm indicates to the isopropyl protons and amide protons, respectively. The significant resonance signal at δ1.5 ppm and δ2.7 ppm indicates to the copolymer including pNIPAAm and GMA segments in the pNIPAAm-co-GMA-Lys HG (Figure 1A). Furthermore, the Lys-functionalized pNIPAAm-co-GMA-Lys HG sample showed the resonance peaks at δ1.8 ppm and the resonance signals at δ3.1 ppm for the hydroxyl –O–H groups and δ2.08 ppm for the amine –N–H groups, confirming that the Lys groups were functionalized in the pNIPAAm-co-GMA-Lys HG system (Figure 1B) [30].

The structure of the pNIPAAm-co-GMA and pNIPAAm-co-GMA-Lys HG samples was determined using FTIR spectroscopy. Figure 1C depicts the FTIR spectrum of a pNIPAAm-co-GMA copolymer with propyl carbon stretching peaks at 2786 cm^−1^ and 2939 cm^−1^, and the stretching vibration mode at 1470 cm^−1^ for the amine (–N–H) groups indicates the existence of NIPAAm segments in the pNIPAAm-co-GMA copolymer [31]. Moreover, an additional vibration band arose at 1381 cm^−1^ for the alkyl C–O–C modes for the epoxy groups, indicating the presence of GMA segments in the pNIPAAm-co-GMA copolymer samples. Additionally, after the Lys units were modified, new peaks formed at 1552 cm^−1^ and 1632 cm^−1^ signifying amine (–NH), carboxylic acid groups, indicating the existence of functionalized Lys units in the pNIPAAm-co-GMA HG sample [32]. Moreover, the FITC conjugation was verified by the appearance of peaks at 1456 cm^−1^ and 1725 cm^−1^, which indicate the imine (–C=N) and –C=S groups of the FITC molecules, respectively (Figure 1C). The pNIPAAm-co-GMA copolymer and the Lys-modified and FITC-conjugated pNIPAAm-co-GMA-Lys HG were successfully synthesized and verified by the FTIR data. SEM analysis was used to observe the surface morphology and particle size of the powder copolymer samples, such as the pNIPAAm-co-GMA and pNIPAAm-co-GMA-Lys HG. As seen in Figure 1D, the pNIPAAm-co-GMA copolymer exhibited micro-size particles with a porous structural morphology. Additionally, the Lys-modified pNIPAAm-co-GMA-Lys HG sample had an aggregated particle with a slightly rough surface morphology (Figure 1E). The establishment of hydrogen bonding interactions between the polymer chains in the pNIPAAm-co-GMA-Lys HG might be the cause of the aggregation.

The surface charge of the pNIPAAm-co-GMA copolymer and pNIPAAM-co-GMA-Lys HG samples was evaluated using a zeta potential analysis at varied pH (pH 7.4, 6.2, and 4.0). The pNIPAAm-co-GMA copolymer and pNIPAAM-co-GMA-Lys HG samples exhibited pH-responsive behavior due to the presence of amine (–NH_2_), amide (–C=O–NH), and carboxylic acid (–COOH) groups.

**Figure 2:**
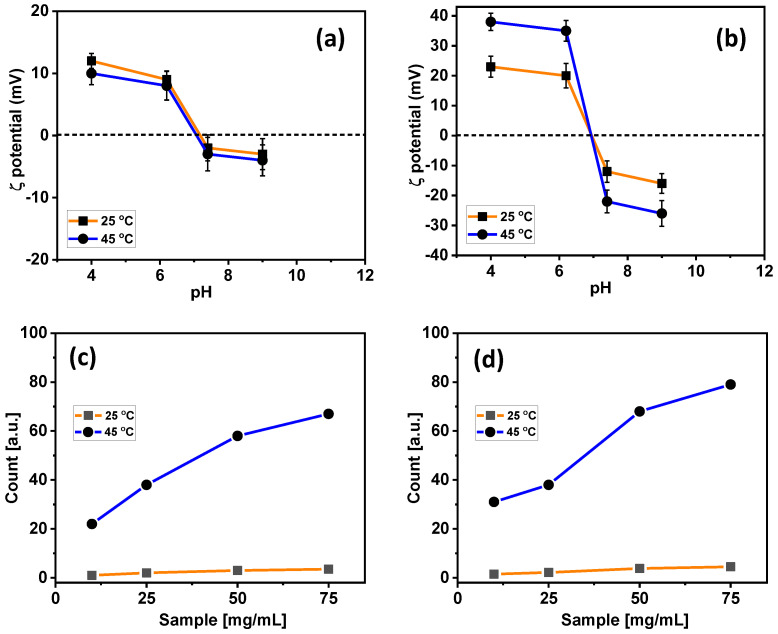
Zeta potential of the (**a**) pNIPAAm-co-GMA copolymer and (**b**) pNIPAAM-co-GMA-Lys HG samples. Mean with error bar *n* = 3. Particle size of (**c**) pNIPAAm-co-GMA copolymer and (**d**) pNIPAAM-co-GMA-Lys HG, respectively, at below and above LCST.

The positive zeta potential values of the pNIPAAm-co-GMA copolymer sample ranged from +12 mV to −3.4 mV at 25 °C, and the zeta potential values of the pNIPAAm-co-GMA copolymer sample at 45 °C showed about +10.2 mV to −5.1 mV as the medium pH, increased from pH 4.0 to pH 9.0 (Figure 2a). The Lys-modified pNIPAAM-co-GMA-Lys HG sample, on the other hand, revealed higher positive zeta potential values of about +24 mV to −15.1 mV at 25 °C, and the zeta potential values were considerably increased to about + 37.3 mV to −26.2 mV in the pH range of pH 4.0 to pH 9.0 and a medium temperature of 45 °C. Moreover, the presence of negatively charged carboxylic acid groups at pH 9 resulted in a negative zeta potential value of about −26.3 mV (Figure 2b). The zeta potential results support that the functionalized Lys groups were presented in the pNIPAAM-co-GMA-Lys HG sample [33].

A dynamic light scattering (DLS) analysis was used to determine the temperature-responsive phase transition, such as the linear-to-globule shape at different temperatures and the optimum particle size. Figure 2c shows that the pNIPAAm-co-GMA copolymer sample exhibited a low DLS intensity at 25 °C and a sample concentration of 20 mg/mL in deionized water. The particle size, on the other hand, rose when the suspension’s medium temperature was raised to 45 °C. This might be because the pNIPAAm-co-GMA copolymer undergoes a phase transition and the linear polymer chains are converted into coil-like globule structures at higher temperatures, resulting in large particles in the solution. Additionally, the Lys-modified pNIPAAM-co-GMA-Lys HG sample revealed considerably larger particle sizes at higher temperatures (45 °C) (Figure 2d), which might be attributed to the temperature-responsive phase transition and formation of hydrogen bonding interactions between the amine and carbonyl groups in the polymer chains in the pNIPAAM-co-GMA-Lys HG samples.

**Figure 3:**
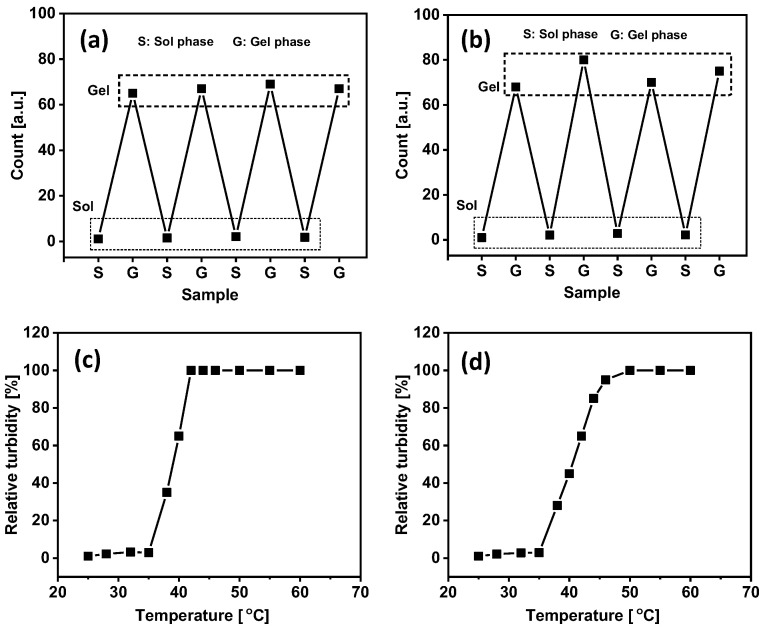
Swelling–deswelling behavior of (**a**) pNIPAAm-co-GMA copolymer; (**b**) pNIPAAm-co-GMA-Lys HG. Relative turbidity of (**c**) pNIPAAm-co-GMA copolymer and (**d**) pNIPAAm-co-GMA-Lys HG, respectively, at 25 °C to 60 °C.

**Scheme 1:**
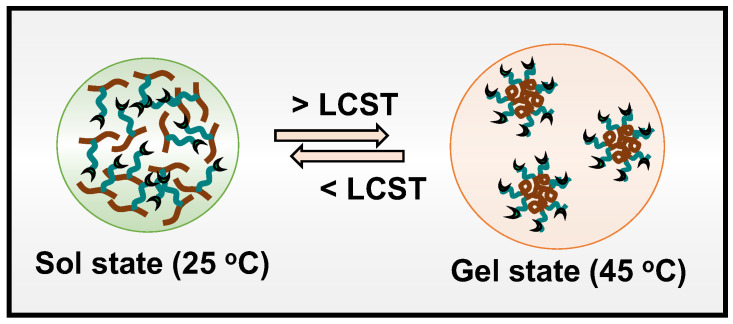
Represent the phase transition of pNIPAAm-co-GMA-Lys HG system under below and above LCST condition in the presence of temperature stimuli.

The swelling–deswelling and temperature-responsive phase transitions, as well as the relative turbidity of the pNIPAAm-co-GMA-Lys HG sample, were studied by using a dynamic light scattering (DLS) analysis. At various temperatures, the pNIPAAm-co-GMA-Lys HG solution (20 mg/mL) in deionized water was measured (25 °C and 45 °C). Figure 3a shows that the pNIPAAm-co-GMA HG sample had no considerable particles produced at 25 °C, whereas a noticeable phase transition occurs, and micro-size particles were formed when the pNIPAAm-co-GMA HG solution was measured at 45 °C. Similar phenomena were observed for the Lys-modified pNIPAAm-co-GMA-Lys HG (Figure 3b). The observed results were caused due to the temperature-induced phase transformation of the linear chain structure into a coil-like globule structure at the above LCST of the pNIPAAm-co-GMA-Lys HG sample (Scheme 1) [34]. These findings support that the pNIPAAm-co-GMA HG undergoes a temperature-responsive phase transition at the above LCST.

Furthermore, the relative turbidity of the pNIPAAm-co-GMA HG and pNIPAAm-co-GMA-Lys HG samples, respectively, were investigated using UV-vis spectral analysis at temperatures ranging from 25 °C to 60 °C. At temperatures below the LCST (<40 °C), the pNIPAAm-co-GMA HG and pNIPAAm-co-GMA-Lys HG samples showed a homogenous and clear transparent solution, and no considerable absorption, as shown in Figure 3c,d. Below the LCST, the pNIPAAm-co-GMA HG and pNIPAAm-co-GMA-Lys HG samples became hydrated by absorbing water molecules and the polymer chains exist in a linear chain structure and the solution appears transparent. On the other hand, the clear solution turns turbid, and the turbidity of the solution increased with increasing the solution temperature above the LCST. At above the LCST, the linear chains of the pNIPAAm-co-GMA HG and pNIPAAm-co-GMA-Lys HG samples collapse and turn into hydrophobic globule micelle-like structures (Figure 3c,d) [35].

### 2.2. Stimuli-Responsive Drug Delivery Behavior of the pNIPAAm-co-GMA-Lys/Cur HG System

A copolymer can be created by combining suitable comonomers, and the resultant copolymer can be further functionalized at the hydrophilic tail’s terminal end. These functional groups in the copolymer structure improve the copolymer’s ability to behave as a nanocarrier. The copolymer structure’s hydrophobic and hydrophilic segments may form a core-shell structure, and the polymeric nanoparticles maintain their structure even at low concentrations below the critical micelle concentration of micelles [36,37]. These copolymer nanocarriers can considerably improve drug targeting to specific regions due to the inclusion of stimuli-responsive functional groups in the copolymer structure. Because of the phase transition and environmental stimuli trigger, the copolymer structure exists in the linear form below the LCST and may accept higher quantities of loaded drugs and release them in a regulated manner above the LCST. The delivery characteristics of the copolymer nanocarriers may vary depending on the polymeric structure. For example, dendrimers, nanospheres, nanocapsules, nanogels, nanomicelles, etc., undergo moderate confirmational changes, employed as drug carriers [38,39]. The ability to undergo obvious phase changes in response to physical stimuli is one of the unique properties of dual-stimuli-responsive hydrogels. The pNIPAAm-co-GMA-Lys HG was synthesized using a pNIPAAm segment including hydrophilic amide (–C=O–NH) and hydrophobic isopropyl groups. Because of the higher LCST of the pNIPAAm-co-GMA-Lys HG sample, the rapid micellar formation may be reduced when injected into the body. The encapsulated drug may be administered selectively and in a controlled manner by altering the temperature stimulation. The sensitivity to the temperature behavior of the pNIPAAm-co-GMA-Lys HG sample suggests that it might be used in temperature-responsive drug delivery applications. To verify this, a model anticancer Cur was loaded into the pNIPAAm-co-GMA-Lys HG by mixing them at low temperatures, and the drug-loaded pNIPAAm-co-GMA-Lys HG was separated easily by increasing the temperature over the LCST.

**Figure 4:**
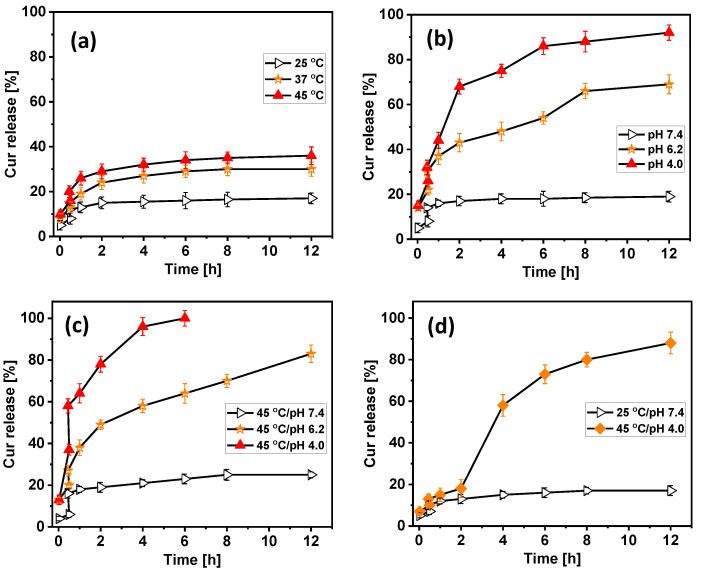
In vitro drug delivery efficiency of pNIPAAm-co-GMA-Lys/Cur HG system. (**a**) Cur release at different temperature conditions, (**b**) Cur release at different pH stimuli, and (**c**) Cur release with the combined temperature and pH conditions. (**d**) Temperature- and pH-triggered drug release behavior after 2 h. Mean with error bar *n* = 3.

Different temperature and pH stimuli conditions, specifically the (i) different temperature (25 °C, 37 °C, and 45 °C), (ii) different pH (pH 7.4, 6.2 and 4.0), and (iii) combined temperature and pH (45 °C/pH 7.4; 45 °C/pH 6.2; and 45 °C/pH 4.0), respectively, were examined to verify the temperature and pH dual-stimuli-responsive drug delivery behavior of the pNIPAAm-co-GMA-Lys HG system. The drug-loaded pNIPAAm-co-GMA-Lys/Cur HG sample’s temperature-sensitive drug release behavior was first investigated. The temperature-sensitive Cur release from the pNIPAAm-co-GMA-Lys/Cur HG system is shown in Figure 4a. In 12 h, approximately ~17%, ~22%, and ~37% of the Cur was released at 25 °C, 37 °C, and 45 °C, respectively (Table 1). A considerable increase in the Cur release (~20%) at 45 °C may indicate a temperature-induced release of physically entrapped drug molecules from the pNIPAAm-co-GMA-Lys/Cur HG owing to the polymer chain phase transition from linear chains to a globular coil state, which induces the entrapped drug molecules to diffuse out from the pNIPAAm-co-GMA-Lys/Cur HG polymer micelles [40].

Second, the drug release behavior of the pNIPAAm-co-GMA-Lys/Cur HG was examined at various pH (pH 7.4, 6.2, and 4.0) levels. Figure 4b showed the Cur release behavior of the pNIPAAm-co-GMA-Lys/Cur HG system at various pH levels. Specifically, approximately ~18% of the Cur was released in 12 h under pH 7.4 conditions, at 25 °C. In comparison, approximately ~68% and over ~90% of the Cur was released in 12 h at pH 6.2 and 4.0 conditions, respectively (Table 2). As can be seen in Figure 4b, the Cur release was higher at acidic pH (pH 6.2 and 4.0) than at pH 7.4. This could be explained by the protonation of the drug-binding sites such as the amine, carbonyl, and hydroxyl groups present in the pNIPAAm-co-GMA-Lys/Cur HG system under acidic pH conditions, which resulted in a significantly enhanced Cur release. Under acidic pH conditions, an electrostatic repulsive force is anticipated between protonated drug molecules and drug-binding sites, causing the drug molecules to be released from the pNIPAAm-co-GMA-Lys/Cur HG system [41].

The cumulative drug release behavior of the pNIPAAm-co-GMA-Lys/Cur HG system under the combined temperature and pH settings was investigated. As shown in Figure 4c, a very slow release of Cur was observed, with approximately ~22% of the Cur release occurring in 12 h at 45 °C/pH 7.4. Because of the slow release of the Cur caused by a temperature-induced phase transition, only about ~22% of the Cur was released at 45 °C/pH 7.4 conditions. These results revealed that drug molecules and drug-binding functional groups have substantial electrostatic and/or hydrogen bonding interactions in the pNIPAAm-co-GMA-Lys/Cur HG system at 45 °C/pH 7.4. The Cur release, on the other hand, was dramatically enhanced, noticeably by about ~83% in 12 h under the 45 °C/pH 6.2 conditions. Further, the Cur release was significantly increased, and an almost complete release occurred in 6 h at higher temperatures and increased pH settings, such as at the 45 °C/pH 4.0 conditions (Figure 4c) (Table 2). A complete drug release was achieved under the 45 °C/pH 4.0 conditions, which might be attributed to the dual stimuli effects such as the temperature-induced phase transition and pH-induced protonation of drug-binding sites, namely the amine, hydroxyl, and carboxylic acid groups present in the pNIPAAm-co-GMA-Lys/Cur HG system and drug molecules, in particular, having a significant impact in releasing the loaded drug molecules from the pNIPAAm-co-GMA-Lys/Cur HG system [42]. Moreover, the temperature- and acidic pH-triggered drug release behavior of the pNIPAAm-co-GMA-Lys/Cur HG system was evaluated by examining the Cur release at the 25 °C/pH 7.4 conditions for the initial 2 h, and then the temperature and medium pH were increased to 45 °C/4.0 and the released drug was determined. As shown in Figure 4d, the slow drug release of about ~18% was observed at 25 °C/pH 7.4, whereas after altering the release medium condition to 45 °C/pH 4.0, the drug release was noticeably increased and reached over ~80% in 12 h. The drug release experimental findings demonstrated that the produced pNIPAAm-co-GMA-Lys/Cur HG system is more efficient for dual temperature- and pH-stimuli-responsive drug delivery than the single stimuli, such as only a pH- or a temperature-stimuli-responsive drug release [43].

### 2.3. Cell Viability Study

**Figure 5:**
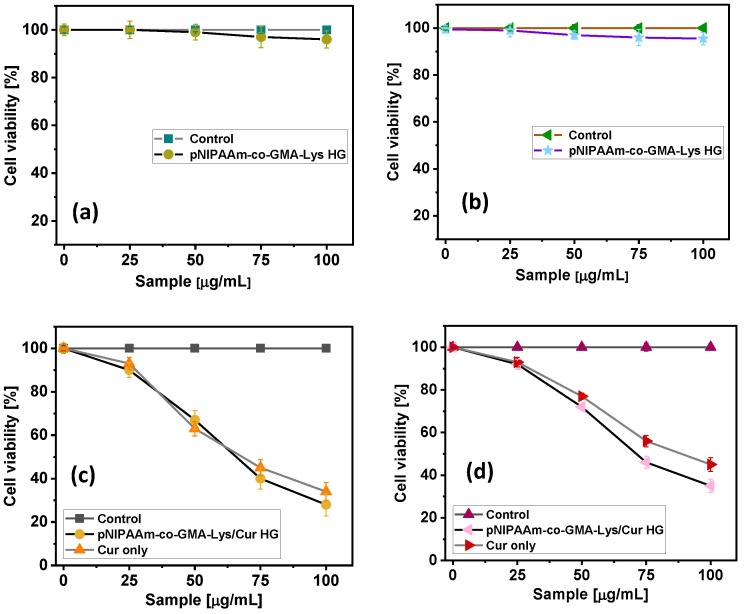
(**a**) In vitro cytocompatibility of only pNIPAAm-co-GMA-Lys HG-treated (**a**) MDA-MB-231 cells and (**b**) HepG2 cells. In vitro cytocompatibility of drug-loaded pNIPAAm-co-GMA-Lys/Cur HG- and pure Cur drug-treated (**c**) MDA-MB-231 cells and (**d**) HepG2 cells, respectively, at different concentrations. Statistical significance to the cell toxicity with different samples (* significant *p* < 0.05).

MDA-MB-231 cells were used to examine the cell viability of the pNIPAAm-co-GMA-Lys HG, pNIPAAm-co-GMA-Lys/Cur HG, and pure Cur drug, respectively. As shown in Figure 5a,b, the pNIPAAm-co-GMA-Lys HG system demonstrated over ~90% and ~92% viability to the MDA-MB-231 cells and HepG2 cells at the tested sample doses, indicating that the pNIPAAm-co-GMA-Lys HG is highly cytocompatible. The drug-encapsulated pNIPAAm-co-GMA-Lys/Cur HG, on the other hand, exhibited a sample concentration-dependent cell toxicity to the MDA-MB-231 cells and HepG2 cells (Figure 5c,d) [44]. The MTT assay findings corroborate the notion that the pNIPAAm-co-GMA-Lys HG system might be effective for dual temperature- and pH-stimuli-responsive anticancer drug delivery to target regions.

### 2.4. Fluorescence Image Analysis

Bioimaging is considered to be a vital topic in the biomedical field for diagnosis. It is worthwhile to create a fluorescent hydrogel to expand the applications of polymeric hydrogels beyond drug delivery. As a result, we chose the FITC molecule to conjugate with the pNIPAAm-co-GMA-Lys HG that we had synthesized.

**Figure 6:**
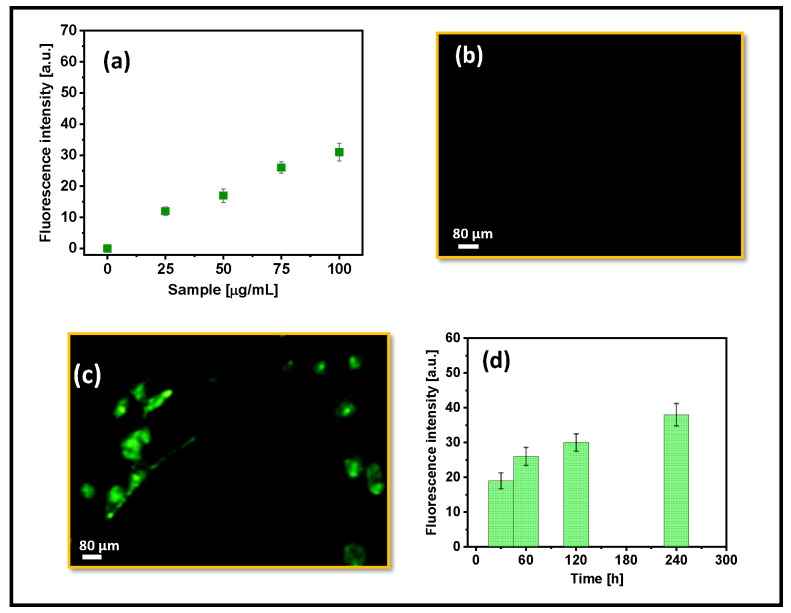
(**a**) Fluorescence intensity of FITC-conjugated pNIPAAm-co-GMA-Lys HG at different sample concentrations. Fluorescence microscopy images of MDA-MB-231 cells represent (**b**) control sample and (**c**) pNIPAAm-co-GMA-Lys HG-treated cells. (**d**) Green fluorescence signal intensity of pNIPAAm-co-GMA-Lys HG system, at different incubation times.

The FITC molecules were covalently linked by the chemical interaction between the thioisocyanate and the amine groups present in the Lys functionalities. The fluorescence was caused by the transfer of the fluorescence resonance energy between the thiourea and FITC molecules [45]. To test the fluorescence behavior of the FITC-conjugated pNIPAAm-co-GMA-Lys HG system, the sample at different concentrations (0, 25, 50, 75, and 100 µg/mL), respectively, was exposed to MDA-MB-231 cells and the green fluorescence signals were evaluated. As shown in Figure 6a, the fluorescence signal intensity was increased with increasing sample concentrations which was due to the greater number of FITC molecules conjugated with the polymer chains in the pNIPAAm-co-GMA-Lys HG system. Further, fluorescence microscopy was then used to visualize the FITC-induced green fluorescence of the pNIPAAm-co-GMA-Lys HG. As shown in Figure 6b, no fluorescence was observed in the control cells; however, the pNIPAAm-co-GMA-Lys HG sample-exposed cells showed green fluorescence (Figure 6c). The green fluorescence signals seen in the MDA-MB-231 cells demonstrated that the pNIPAAm-co-GMA-Lys HG was taken up by the cells and that the fluorescence signal was not significantly quenched under intracellular conditions. Moreover, the intensity of the green fluorescence increased with an increasing incubation time (Figure 6d), demonstrating that more of the pNIPAAm-co-GMA-Lys HG can be internalized into MDA-MB-231 cells, implying that the pNIPAAm-co-GMA-Lys HG could potentially be employed in green fluorescence-based bioimaging applications.

## 3. Conclusions

In this study, we synthesized a pNIPAAm-co-GMA-Lys copolymer hydrogel system for dual-stimuli-responsive drug delivery applications, for stimuli such as temperature and pH. Cur, an anticancer drug, is used as a model cargo to validate the drug loading and stimuli-responsive drug release behavior of the pNIPAAm-co-GMA-Lys HG system. The existence of amine and carboxylic acid groups in the functionalized Lys units in the pNIPAAm-co-GMA-Lys HG resulted in a high drug loading content of around ~69%. The in vitro drug release experiments show that the pNIPAAm-co-GMA-Lys/Cur HG exhibits almost a complete drug release under combined temperature and pH (45 °C/pH 4.0) conditions. The MTT assay results further show that the pNIPAAm-co-GMA-Lys HG is cytocompatible which could be suitable in drug delivery applications. Furthermore, because of the presence of FITC molecules conjugated with the pNIPAAm-co-GMA-Lys HG, it could be used for green fluorescence-based bioimaging applications. According to the results of the experiments, it is reasonable to conclude that the synthesized pNIPAAm-co-GMA-Lys HG could potentially serve dual temperature- and pH-stimuli-responsive drug delivery and bioimaging applications.

## 4. Chemicals 

N-Isopropylacrylamide (NIPAm, 97%), glycidyl methacrylate (GMA, 97%), 2,2-azobisisobutyronitrile (AIBN, 12 wt.% in acetone), fluorescein isothiocyanate (FITC, 95%), tetrahydrofuran (THF, 99.9%), hexane (95%), and curcumin (98%) were purchased from Sigma Aldrich Chemical Co., Saint Louis, MO, USA, and used as received.

### 4.1. Preparation of pNIPAAm-co-GMA-Lys HG System

**Scheme 2:**
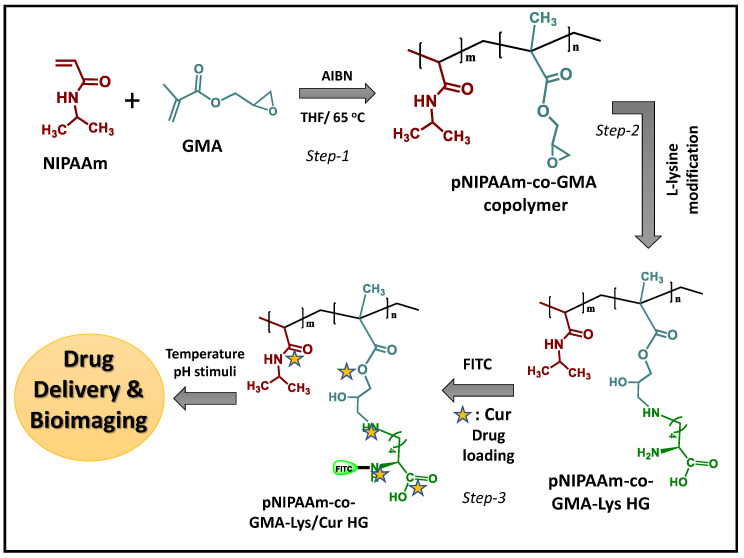
Represents the synthesis of pNIPAAm-co-GMA copolymer (Step-1); L-lysine modified pNIPAAm-co-GMA-Lys HG system (Step-2); and FITC conjugation and drug loading in the pNIPAAm-co-GMA-Lys HG (Step-3).

In the first step, the pNIPAAm-co-GMA copolymer was synthesized as follows. For this synthesis, about 3.0 g (26.2 mmol) of NIPAAm monomer was taken into a 100 mL reaction flask and dissolved in 50 mL THF solvent. To this, about 4.2 g (26.5 mmol) of GMA was added and the reaction solution was purged with nitrogen gas for 30 min. Further, approximately 0.1 g AIBN was added to initiate the reaction and the reaction flask was magnetically stirred under inert conditions at 65 °C for 24 h [46]. The resulting viscous mass was precipitated in a beaker containing 250 mL hexane and the precipitation process was repeated 3 times to eliminate the unreacted monomer molecules. The obtained precipitate was dried at room temperature under vacuum, overnight. The obtained product was labeled as pNIPAAm-co-GMA copolymer (Scheme 2, Step-1).

In the second step, the L-Lysine (Lys) was conjugated with the pNIPAAm-co-GMA copolymer by reacting the amine group of Lys units with the epoxy groups of GMA segments by the ring-opening reaction. To perform this conjugation, about 1.0 g of the synthesized pNIPAAm-co-GMA copolymer was placed in a 100 mL reaction flask containing THF solvent (50 mL). To this, about 0.4 g (2.7 mmol) of Lys (dissolved in 5 mL methanol) was added and the reaction mixture was kept for reaction at 60 °C for 24 h [47]. After the reaction was completed, the existing solvent was removed by a rotary evaporator and the resulting product was precipitated in a hexane:methanol (10:2) mixture to remove the unreacted Lys molecules. The resulting product was labeled as the pNIPAAm-co-GMA-Lys copolymer (Scheme 2, Step-2).

In the third step, the fluorophore FITC was conjugated by reacting the FITC molecules with the amine groups present in the Lys units of the pNIPAAm-co-GMA-Lys copolymer [48]. For this conjugation, about 0.5 g of the pNIPAAm-co-GMA-Lys copolymer was dissolved in methanol (50 mL) and approximately 0.5 mL of the FITC solution (0.1 mg/mL) was added and the reaction was performed at 50 °C for 12 h under a dark condition. Then, the resulting product was precipitated in a 200 mL hexane: methanol (8:2) mixture. The resulting FITC-conjugated product was dried at room temperature under vacuum, overnight (Scheme 2, Step-3).

### 4.2. Characterization

The ^1^H-NMR instrument (OXFORD, 600 MHz, Concord, MA, USA) was utilized to confirm the synthesis of the pNIPAAm-co-GMA-Lys copolymer. Scanning electron microscopy (SEM, JEOL 6400, 10 kV, North Billerica, MA, USA) was used to observe the surface morphology. A Fourier-transform infrared (FTIR) analysis was carried out by the KBr pelleting method using the JASCO FTIR 4100 instrument (Easton, MD, USA). A surface charge and particle size analysis were performed using Malvern Zetasizer Nano-ZS (Malvern, UK). A UV-visible spectral analysis was measured using a UV-vis spectrophotometer (Agilent Inc. Model 8453, Santa Clara, CA, USA).

### 4.3. Turbidity Measurement

To measure the turbidity of the pNIPAAm-co-GMA-Lys HG samples, the copolymer with a sample concentration of 25 mg/mL in deionized water was prepared. The absorbance of the prepared pNIPAAm-co-GMA-Lys HG solution at a different temperature range from 25 °C to 60 °C was measured using UV-vis spectral analysis.

### 4.4. Drug Loading and Release Behavior of the pNIPAAm-co-GMA-Lys HG

A model anticancer drug, curcumin, was used to evaluate the drug loading and release from the drug-loaded pNIPAAm-co-GMA-Lys HG sample. About 10:4 wt.:wt. ratio of polymer: the drug was used for loading Cur into the pNIPAAm-co-GMA-Lys HG via the swelling diffusion technique [49]. For this, about 0.2 g of the pNIPAAm-co-GMA-Lys HG sample was solubilized in 5 mL deionized water and then the Cur solution (20 mg/mL) was added, and the suspension was magnetically stirred at room temperature, overnight. The Cur-loaded sample was separated at 50 °C and the resulting supernatant was used to estimate the drug loading content into the pNIPAAm-co-GMA-Lys HG. The loaded Cur was determined by using UV-vis spectral analysis at 430 nm. The Cur loading into the pNIPAAm-co-GMA-Lys/Cur HG was estimated by applying the following equation. Drug loading content (%) = [(Wt. of loaded drug)/(Wt. of drug in sample)] × 100. The estimated Cur loading into the pNIPAAm-co-GMA-Lys/Cur HG was approximately ~69%. Similarly, the drug loading efficiency of the pNIPAAm-co-GMA-Lys HG was determined as follows. Drug loading efficiency (%) = [Wt.of loaded drug/Wt. of feeding drug] × 100. The drug loading efficiency was estimated to be ~70%.

The in vitro drug release experiments were carried out at different conditions, such as (i) different temperatures (25 °C, 37 °C, and 45 °C); (ii) different pH (pH 7.4, 6.2, and 4.0); and (iii) the combined temperature and pH (45 °C/pH 7.4 and 45 °C/pH 4.0). For the drug release study, the pNIPAAm-co-GMA-Lys/Cur HG sample (50 mg/mL) was taken in a dialysis bag (Mol. wt. cut off 8000 kDa) and put into a 50 mL beaker containing the phosphate buffer saline (PBS) solution. The beaker was placed onto a magnetic stirrer and the medium pH and temperature were maintained appropriately. About 1.0 mL of the release medium was extracted from the beaker at the prefixed time point and the released drug molecules were determined by UV-vis spectrometer at 430 nm. The drug release was determined as follows. Cumulative drug release (%) = (Mass of Cur release at time t/Total mass of Cur in the HG sample) × 100.

### 4.5. Biocompatibility Study

The biocompatibility of the synthesized pNIPAAm-co-GMA-Lys HG, Cur-loaded pNIPAAm-co-GMA-Lys/Cur HG, and pure Cur drug, respectively, were evaluated by the 3-(4,5-dimethylthiazol-2-yl)-2,5-diphenyl tetrazolium bromide (MTT) assay analysis. To carry out this experiment, MDA-MB-231 cells and HepG2 cells, respectively, (2 × 10^4^ cells/well), were cultured into a 96-well plate at 37 °C for 24 h. Next, the fresh medium containing various concentrations of the pNIPAAm-co-GMA-Lys HG, Cur-loaded pNIPAAm-co-GMA-Lys/Cur HG, and pure Cur drug, respectively, were added and further incubated for 6 h. Further, the MTT solution was added to each well and incubated for 3 h. Finally, the formed purple crystal was solubilized by adding a fresh cold dimethyl sulfoxide (20 μL/mL) and the absorbance was measured at 590 nm. Cell viability (%) = [(OD_treated_)/(OD_control_)] × 100. OD_treated_: cells treated with pNIPAAm-co-GMA-Lys/Cur HG. OD_control_: cells treated with pNIPAAm-co-GMA-Lys HG.

### 4.6. Fluorescence Imaging Study

The cell uptake behavior of the FITC-conjugated pNIPAAm-co-GMA-Lys HG was determined by treating with MDA-MB-231 cells and observed using a fluorescence microscope technique. For this study, the FITC-conjugated pNIPAAm-co-GMA-Lys/Cur HG (5 mg/mL) was treated with MDA-MB-231 cells and incubated for 4 h. Then, the sample-treated cells were fixed with paraformaldehyde (4%) for 20 min. After 20 min incubation, the cells were washed with fresh cold PBS buffer, and the cells were visualized using a fluorescence microscope.

### 4.7. Statistical Analysis

All results, expressed as the mean ± SD, were analyzed using a two-tailed Student’s *t*-test or one-way analysis of variance (ANOVA). The acceptable level of significance was *p* < 0.05.

## Figures and Tables

**Table 1:** Drug release behavior from pNIPAAm-co-GMA-Lys/Cur HG system at different temperatures.

Sample	LCST (°C)	Drug Release (%)		
		25 °C	37 °C	45 °C
pNIPAAm-co-GMA-Lys/Cur HG	~38–40	17	22	37

**Table 2:** Drug release efficiency from pNIPAAm-co-GMA-Lys/Cur HG system at different pH values.

pH	Drug Release (%)
7.4	18
6.2	68
4.0	90
45 °C/7.4	22
45 °C/6.2	83
45 °C/4.0	100
